# AEG-1 is associated with hypoxia-induced hepatocellular carcinoma chemoresistance via regulating PI3K/AKT/HIF-1alpha/MDR-1 pathway

**DOI:** 10.17179/excli2016-694

**Published:** 2016-11-30

**Authors:** Yong Xie, De-Wu Zhong

**Affiliations:** 1 Department of Hepatobiliary Surgery, the 2nd XiangYa Hospital of Centre South University, 139#, Renmin Road, Changsha, Hunan, P.R. China

**Keywords:** AEG-1, hypoxia, hepatocellular carcinoma, chemoresistance, PI3K/AKT/HIF-1alpha/MDR-1 pathway

## Abstract

Hypoxia is a common characteristic of hepatocellular carcinoma (HCC) associated with reduced response to chemotherapy, thus increasing the probability of tumor recurrence. Astrocyte elevated gene-1 (AEG-1) has been involved in a wide array of cancer progression including proliferation, chemoresistance, angiogenesis and metastasis, but its effect on HCC chemoresistance induced by hypoxia is unclear. In this study, expression of AEG-1 and multiple drug resistance (MDR-1) were examined in HCC using immunohistochemical staining and RT-PCR. Furthermore, their expression levels were detected in HCC HepG2 cells in normoxia or hypoxia via RT-PCR and Western blot assays. Specific shRNAs were used to silence AEG-1 expression in HepG2 cells. Results showed AEG-1 and MDR-1 expression were higher in HCC tissues than in adjacent normal tissues. Incubation of HepG2 cells in hypoxia increased expression of AEG-1 and MDR-1, compared to incubation in normoxia. Exposure to hypoxia blunted sensitivity of HepG2 cells to Adriamycin, 5-fluorouracil and cis-platinum, as evidenced by modest alterations in cell viability and apoptosis rate, however the sensitivity was elevated with AEG-1 knockdown. PI3K/AKT/HIF-1/MDR-1 pathway was attenuated following AEG-1 knockdown in hypoxia. Based on these data, it was suggested that AEG-1 is associated with hypoxia-induced hepatocellular carcinoma chemoresistance via regulating PI3K/AKT/HIF-1/MDR-1 pathway. This study uncovered a novel potential target for development of an effective therapy against hypoxia-induced HCC chemoresistance.

## Introduction

Hepatocellular carcinoma (HCC) is a frequently occurring primary cancer, representing one of the leading cause of cancer-related deaths worldwide (Xu et al., 2014[27]). Of note, HCC has very high prevalence in China occupying half of the cases and deaths (Xu et al., 2014[27]). The Cancer Country Profiles from World Health Organization estimated that 394,770 of new liver cancer cases and 380,772 cancer deaths were occurred in China only in 2014 (Zhang et al., 2016[32]). Chemotherapeutic regimens remain the treatment of choice for hepatocellular carcinoma especially at the advanced stages. Anti-cancer agents including Adriamycin (ADM), 5-fluorouracil (5-FU) and cis-platinum (DDP) are commonly used in the prescriptions to HCC patients in China due to their inexpensive price and easy accessibility (Li et al., 2016[17]). But these chemotherapy options ultimately yield poor outcomes in these patients mainly resulted from the chemoresistant development of HCC. 

It has been identified that the hypoxic tumor microenvironment plays a prominent role in the induction of chemoresistance. Hypoxia is a common characteristic of HCC and other solid tumors (Zhang et al., 2016[32]). Rapid growth of tumors easily leads to insufficient blood supply in the central region of the tumor (Bogaerts et al., 2015[2]). When tumor cells are located further than 100-180 μm from a functional blood vessel, oxygen is difficult to diffuse beyond this distance (Flamant et al., 2012[7]). Besides, fibrogenesis during the development of cirrhosis destroys the normal blood supply to both normal liver tissues and HCC, which can lead to local hypoxia (Bogaerts et al., 2015[2]). Hypoxic environment triggers a variety of adaptive responses in HCC to survival in harsh conditions, and it provides a strong selective pressure for the survival of HCC, which results in the so called “survival of the fittest” and elimination of the inferior (Zhang et al., 2016[32]; Flamant et al., 2012[7]; Bogaerts et al., 2015[2]). Significant evidence indicates that HCC cells that are survival in hypoxia are more resistant to chemotherapy than the cells growing in normoxia (Zhang et al., 2016[32]; Flamant et al., 2012[7]; Bogaerts et al., 2015[2]). A few of signaling molecules are activated by hypoxia and are responsible for the chemoresistance. 

Transcriptional factor hypoxia-inducible factor 1 (HIF-1α) is a master regulator upon hypoxia. HIF-1α is rapidly degraded by proteasome pathway under normoxia, but it becomes stable under hypoxia and translocates to the nucleus controlling its target genes via binds to hypoxia response elements (Suhara et al., 2015[24]). As a target of HIF-1α, WSB-1 is a key protein involved in DNA damage response (Tong et al., 2013[26]). HIF-1α suppresses etoposide-induced cell death in hypoxic HCC cells via regulating WSB-1 (Tong et al., 2013[26]). Suppression of the accumulation and activity of HIF-1α reversely sensitizes HCC to anti-tumor agents, like camptothecin and sorafenib (Cai et al., 2014[3]). Besides, it is found that HIF-1α/MDR-1 (multiple drug resistance) pathway confers chemoresistance to cisplatin in bladder cancer and to doxorubicin in breast cancer upon hypoxic environment (Doublier et al., 2012[5]; Li et al., 2015[18]; Sun et al., 2016[25]). MDR-1 is closely linked to drug resistance through pumping out extensively anti-cancer drugs from cancer cells (Hyuga et al., 2012[9]). These data indicate HIF-1α functions as an essential regulator leading to the development of tumor chemoresistance in hypoxia. Under hypoxic conditions, the serine/threonine kinase Akt is also activated in a wide variety of cancers and involved in mechanisms underlying hypoxia-induced chemoresistance (Lau et al., 2009[11]). AKT acts as a nexus-signaling molecule in the phosphatidylinositol 3-kinase (PI3K)/AKT pathway that is implicated in the regulation of HIF-1α transcriptional activity (Lau et al., 2009[11]). In a HCC xenograft model, blockage of PI3K/AKT/HIF-1α signaling enforces the therapeutic efficacy of hypoxic chemotherapy (Jiao and Nan, 2012[10]). 

Astrocyte elevated gene-1 (AEG-1) was originally cloned as neuropathology-associated gene in primary human embryos astrocytes in 2002 (Su et al., 2002[23]). Further research reveals the dominant role in the development and progression of diverse cancers (Meng et al., 2013[20]; Chang et al., 2016[4]). AEG-1 as a multi-functional protein has been involved in a wide array of cancer progression including pro-survival, chemoresistance, angiogenesis and metastasis, via interaction with multiple oncogenic signaling pathways (Meng et al., 2013[20]; Chang et al., 2016[4]). As for the modulation of chemoresistance, AEG-1 overexpression has been associated with cisplatin resistance in patients with stage III-IV serous ovarian carcinoma and the poor prognosis (Li et al., 2012[14]). However, it is unclear whether AEG-1 participates in the promoting effects of hypoxia on HCC chemoresistance. A close association of AEG-1 with PI3K/AKT pathway has been unveiled in numerous studies (Meng et al., 2013[20]). It has been confirmed that AEG-1 is a downstream target of PI3K/AKT signal because induction of AEG-1 was attenuated by treatment with the PI3K inhibitor LY294002 or overexpression of phosphatase and tensin homolog (PTEN) that is well-known for its inhibitory effects on PI3K/AKT signal (Lee et al., 2006[12]). But, PI3K/AKT signal is also under the regulation of AEG-1. It was reported that AEG-1 overexpression inhibits serum starvation-induced apoptosis by activating the PI3K/AKT signaling pathway (Lee et al., 2006[12]). AEG-1 was found to physically interact with AKT2, resulting in activation of AKT signal and aggression of glioblastoma (Emdad et al., 2015[6]). Through controlling PI3K/AKT signal, AEG-1 is probably implicated in the regulation of HIF-1α/MDR-1 and hypoxia-induced HCC chemoresistance. Our study herein confirmed that AEG-1 represents a propitious therapeutic target for counteracting hypoxia-induced HCC chemoresistance. 

## Materials and Methods

### Tissue samples and immunohistochemical staining

22 pairs of HCC and adjacent normal tissues were obtained from patients, who experienced surgery at the Third Xiangya Hospital of Central South University. The tissues were either embedded in paraffin or placed in liquid nitrogen based on different detections. All patients provided written informed consent. The study was approved by the Ethics Committee of the Third Xiangya Hospital of Central South University and followed the Declaration of Helsinki.

AEG-1 and MDR-1 expression were examined in the paraffin-embedded specimens using immunohistochemistry. Tissue sections were dewaxed in xylene and rehydrated using standard procedures. After they were washed in phosphate-buffered saline (PBS) three times for 5 minutes each, antigen retrieval was undertaken using a pressure cooker at 121 °C for 15 min in 0.01 M citrate buffer (pH 6.0). To block any potential non-specific binding of the secondary antibody, sections were incubated in 10 % normal goat serum for 30 min at room temperature. The slides were incubated with a primary antibody against human AEG-1 (dilution 1:200; ab76742, Abcam, Cambridge, UK) and MDR-1 (dilution 1:200; sc-71557, Santa Cruz Biotechnology Inc., CA, USA) overnight at 4 °C. After the slides were washed with PBS, the sections were incubated with a biotin-labeled secondary antibody (Santa Cruz Biotechnology Inc.). Finally, sections were lightly counterstained using hematoxylin.

### Cell culture and treatments

The human HCC HepG2 cells were purchased from the American Type Culture Collection (ATCC, Manassas, VA, USA). HepG2 cells were maintained in RPMI-1640 medium/DMEM (Hyclone Laboratories Inc., Logan, Utah, USA) supplemented with 10 % fetal bovine serum (Hyclone) and 1 % penicillin-streptomycin in a humidified atmosphere of 5 % CO_2_ at 37 °C. The culture medium was replaced every 3 days. 

Hypoxia treatment was carried out by placing the cells in a sealed chamber (Thermo Forma) filled with mixture gases of 1 % O_2_, 5 % CO_2_, and 94 % N_2_. HepG2 cells were incubated under normoxia or hypoxia for 0, 24, 48 and 72 h, before detection of the mRNA and protein expression levels of AEG-1 and MDR-1. To get insight into the regulatory role of AEG-1 in chemoresistance of HCC under hypoxia, AEG-1 expression was silenced in HepG2 cells by transfection with specific shRNA. HepG2 cells with AEG-1 knockdown or not were incubated with doses of ADM, 5-FU and DDP under normoxia or hypoxia for different periods of time. Cell viability and apoptosis assays were conducted to evaluated impact of down-regulated AEG-1 on HCC chemoresistance. 

### Quantitative real-time reverse transcription polymerase chain reaction (RT-PCR) assay

The HCC and adjacent normal tissue samples were retrieved from liquid nitrogen and grinded to tiny particles, before incubation with TRIzol (Takara, Otsu, Shiga, Japan) to isolated mRNA. HepG2 cells that were harvested in 35 mm dishes were also incubated with TRIzol (Takara) to extract mRNA. 500 ng RNA was applied to synthesis first-strand complementary DNA using first-strand cDNA Synthesis Kit (Takara, Otsu, Shiga, Japan). RT-PCR was performed in a mixture composed of SYBR Green (Takara), 1 μl (0.2 μmol/l) of each primer, and 1.6 μl of complementary DNA from RT-PCR samples. The primer sequences were as follows: AEG-1, 5'-CGGTACCCCGGCTG GGTGAT-3' (forward) and 5'-CTCCTCCG CTTTTTGCGGGC-3' (reverse); MDR-1, 5'-GCCGGGAGCAGTC ATCTGTGG-3' (forward) and 5'-ATCCAT TCCGACCTC GCGCT-3' (reverse); GAP DH, 5'-CAAT GACCCCTTCATTGACC-3' (forward) and 5'-GACAAGCTTCCCGTTC TCAG-3' (reverse). The mRNA levels of AEG-1 and MDR-1 were quantified by the Ct values, and GAPDH was set as an internal control. 

Experiments were performed in triplicate. The relative expression levels were evaluated using the ^-ΔΔ^Ct method as described previously (Yang et al., 2014[29]). Each individual experiment was performed three times independently.

### Western blot assay

Total proteins were extracted from the HepG2 cells with RIPA buffer containing proteinase/phosphatase inhibitors (Thermo, Cambridge, MA). Proteins were separated on a 10 % or 12 % SDS-PAGE gel, and then transferred onto a nitrocellulose membrane (Millipore, Bedford, MA). The membranes were incubated with one of the following antibodies: anti-AEG-1 antibody (1:500; ab45338, Abcam), anti-MDR-1 antibody (1:500; sc-5510, Santa Cruz Biotechnology Inc.), anti-PI3K antibody (1:500; ab22653, Abcam), anti-AKT antibody (1:600; #21054, SAB, Shanghai, China), anti-HIF-1α antibody (1:1000; sc-10790, Santa Cruz Biotechnology Inc.), anti-PTEN antibody (1:500; sc-6818, Santa Cruz Biotechnology Inc.) and anti-GAPDH antibody (1:800; sc-365062, Santa Cruz Biotechnology Inc.). After washing, the membranes were incubated with horseradish peroxidase-conjugated secondary antibodies that target mouse IgG (ab97040, Abcam), rabbit IgG (A0545, Sigma, St Louis, MO, USA), or goat IgG (A5420, Sigma) at a 1:2,000 dilution for 2 h at room temperature. Reactive proteins were detected using Pierce Enhanced Chemiluminscent and SuperSignal™ Chemiluminescent substrates (Thermo Fisher Scientific, Inc., Waltham, MA, USA). 

### shRNA knockdown of AEG-1

Three shRNAs (shRNA-746, shRNA-1490 and shRNA-1576) targeting AEG-1 were purchased from GenePharma Co., Ltd (Shanghai, China). Transfection of shRNAs and scrambled siRNA into HepG2 cells were performed using Lipofectamine 2000 (Invitrogen, Carlsbad, CA, USA) according to the manufacturer's instructions. RT-PCR, Western blot analysis and an Olympus Fluorview 1000 confocal microscope were used to select clones with stable knockdown of AEG-1. 

### Cell viability assay

HepG2 cells were seeded in 96-well plates in 100 μL complete medium and allowed to attach overnight. After cells were subjected to different treatments, cell viability was measured using 3-(4,5-dimethyl-2-thiazolyl)-2,5-diphenyl-2H-tetrazolium bromide (MTT) assay. MTT (20 ml) solution was added into wells of 96-well plates for 1 h at 37 °C. Absorbance was recorded at 490 nm using a microplate reader (Bio-Rad, Richmond, CA). 

### Apoptosis assay by flow cytometry

After subjected to different treatments, HepG2 cells were washed and then stained with FITC-conjugated Annexin V and PI (Clontech, Beijing, China) at room temperature for 15 min in the dark. The cells were collected and analyzed by a FACSCanto II flow cytometer (Becton Dickinson Immunocytometry System). 

### Statistics

All values are expressed as mean ± SEM. Differences between groups were analyzed by one-way ANOVA with SPSS 13.0 (IBM, Armonk, NY, USA), followed by Bonferroni post-hoc analyses as appropriate. Graphs were generated with Excel. *P* < 0.05 was denoted as statistically significant.

## Results

### AEG-1 and MDR-1 expression in HCC and adjacent normal tissues

**Figure 1:**
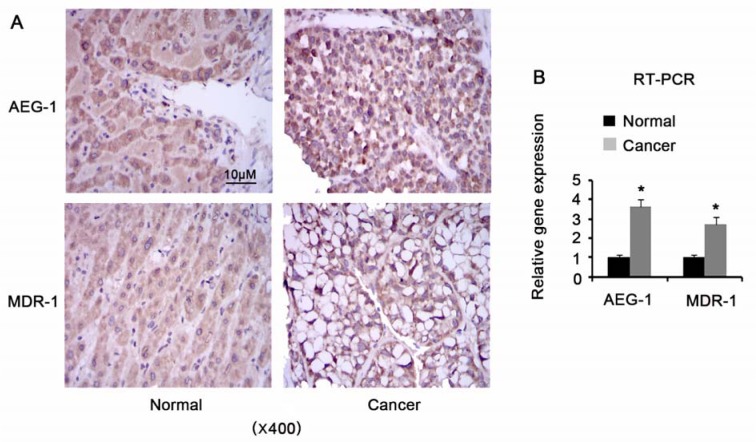
AEG-1 and MDR-1 expression in HCC and adjacent normal tissues AEG-1 and MDR-1 expression in HCC and adjacent normal tissues were investigated by immunohistochemical staining (A) and RT-PCR assays (B). AEG-1: Astrocyte elevated gene-1; MDR-1: multiple drug resistance gene-1; Cancer: Hepatocellular carcinoma; Normal: matched adjacent normal liver tissues. *P < 0.05 vs. control, n = 22.

AEG-1 and MDR-1 expression in HCC and adjacent normal tissues were investigated by immunohistochemical staining and RT-PCR assays. Figure 1A(Fig. 1) shows the representative pictures in immunohistochemical staining assay. It was observed that AEG-1 and MDR-1 were expressed in cytoplasm in normal liver tissues. Similarly, HCC tissues exhibited AEG-1 and MDR-1 expression in cytoplasm. RT-PCR is a useful approach to quantify mRNA expression of targeted genes. With this method, AEG-1 and MDR-1 were found to be up-regulated in HCC tissues compared to adjacent normal tissues (*P* = 0.023 and *P* = 0.039 respectively, Figure 1B(Fig. 1)). 

### Hypoxia triggered up-regulation of AEG-1 and MDR-1 in HCC cells 

**Figure 2:**
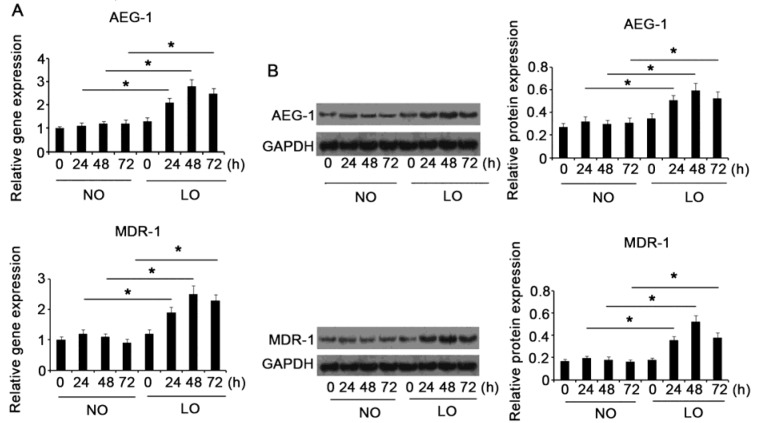
Hypoxia triggered up-regulation of AEG-1 and MDR-1 in HCC cells HCC HepG2 cells were incubated under normoxia or hypoxia for 0, 24, 48 and 72 h, before detection of the mRNA (A) and protein expression (B) levels of AEG-1 and MDR-1. AEG-1: Astrocyte elevated gene-1; MDR-1: multiple drug resistance gene-1; NO: normoxia; LO: low oxygen. *P < 0.05 vs. control, n = 4.

To understand the roles of AEG-1 and MDR-1 in hypoxia-induced chemoresistance in HCC, we initially detected AEG-1 and MDR-1 expression under hypoxic environment via RT-PCR and Western blot assays. HCC HepG2 cells were incubated under normoxia or hypoxia for 0, 24, 48 and 72 h, before detection of the mRNA and protein expression levels of AEG-1 and MDR-1. As Figure 2A(Fig. 2) noted, mRNA expression of AEG-1 was higher in hypoxia than in normoxia at time points of 24 (*P* = 0.038), 48 (*P* = 0.03) and 72 h (*P* = 0.036). Similarly, MDR-1 showed higher mRNA expression in hypoxia for 24 (*P* = 0.042), 48 (*P* = 0.031) and 72 h (*P* = 0.035) compared to that in normoxia. Reflected by Western blotting, AEG-1 showed higher protein expression level in hypoxia than in normoxia at time points of 24 (*P* = 0.033, Figure 2B(Fig. 2)), 48 (*P* = 0.026) and 72 h (*P* = 0.037). Incubation of HepG2 cells in hypoxia for 24 (*P* = 0.035), 48 (*P* = 0.027) and 72 h (*P* = 0.031) resulted in higher MDR-1 protein level compared that in normoxia.

### Knocking down AEG-1 expression in HepG2 cells

**Figure 3:**
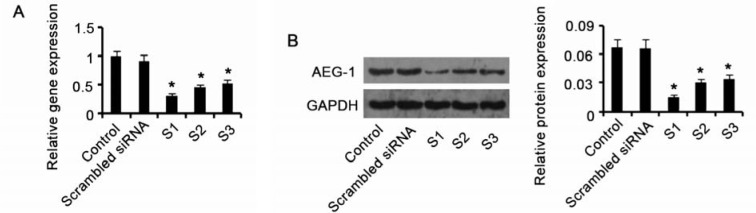
Knocking down AEG-1 expression in HepG2 cells HepG2 cells were transfected with three shRNAs (shRNA-746, shRNA-1490 and shRNA-1576) that specifically target AEG-1 to silence AEG-1 expression. Then the cells were incubated under hypoxia for 48 h. Results obtained from RT-PCR (A) and Western blot (B) assays showed that shRNA-746 had the strongest inhibitory effect on AEG-1 expression level. AEG-1: Astrocyte elevated gene-1. *P < 0.05 vs. control, n = 4.

To silence AEG-1 expression in HepG2 cells, the cells were transfected with three shRNAs (shRNA-746, shRNA-1490 and shRNA-1576) that specifically target AEG-1. Then the cells were incubated under hypoxia for 48 h. Results obtained from RT-PCR assay showed that shRNA-746 had the strongest inhibitory effect on AEG-1 mRNA level (*P* = 0.017 *vs*. control, Figure 3A(Fig. 3)) compared with other shRNAs. Western blot assay indicated that shRNA-746 attenuated AEG-1 protein expression to the lowest level among all these shRNAs (*P* = 0.019 *vs*. control, Figure 3B(Fig. 3)). shRNA-746 was thus used in further study.

### AEG-1 knockdown enhanced responses of HepG2 cells to ADM, 5-FU and DDP under hypoxia

**Figure 4:**
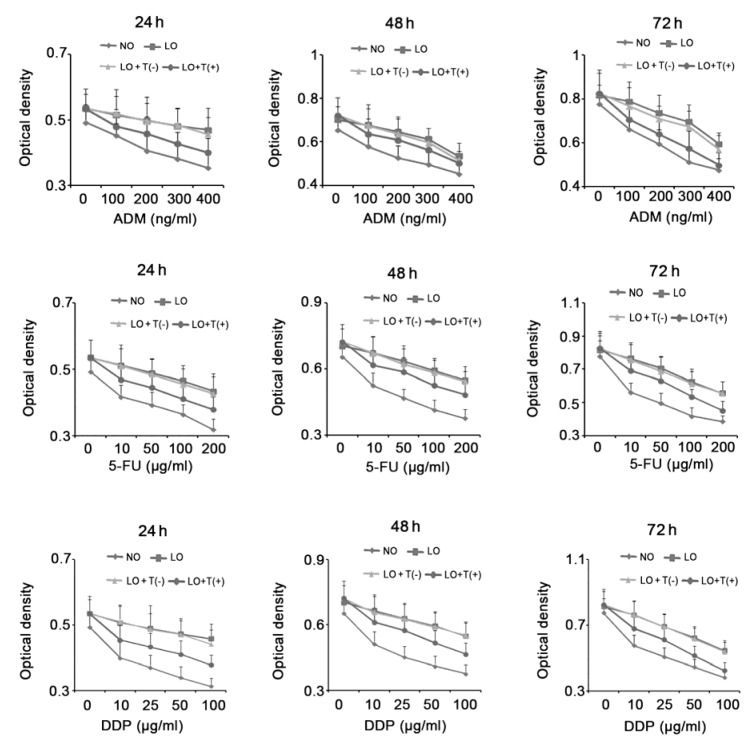
AEG-1 knockdown enhanced cytotoxicity of ADM, 5-FU and DDP to HepG2 cells under hypoxia HepG2 cells with AEG-1 knockdown or not were incubated with doses of ADM, 5-FU and DDP under normoxia or hypoxia for different periods of time. Cell viability was determined by MTT assay. ADM: Adriamycin; 5-FU: 5-fluorouracil; DDP: cis-platinum; AEG-1: Astrocyte elevated gene-1; NO: normoxia; LO: low oxygen; LO + T(-): HepG2 cells transfected with scrambled siRNA were incubated in hypoxia; LO + T(+) : HepG2 cells transfected with shRNA-746 were incubated in hypoxia.

HepG2 cells with AEG-1 knockdown or not were incubated with doses of ADM, 5-FU and DDP under normoxia or hypoxia for different periods of time. Variation curves of cell viability, determined by MTT assay, after the treatments were presented in Figure 4(Fig. 4). It was observed that ADM dose-dependently decreased HepG2 cell viability after the incubation under normoxia for 24, 48 and 72 h. In contrast, incubation in hypoxia for different periods of time (24, 48 and 72 h) attenuated cytotoxic effect of ADM at all treated dosages. But, AEG-1 knockdown to certain extent reversed cytotoxic effect of ADM on HepG2 cells in hypoxia. Hypoxic environment also inhibited cytotoxic effects of 5-FU and DDP, but depletion of AEG-1 enhanced their effects in hypoxia. 

**Figure 5:**
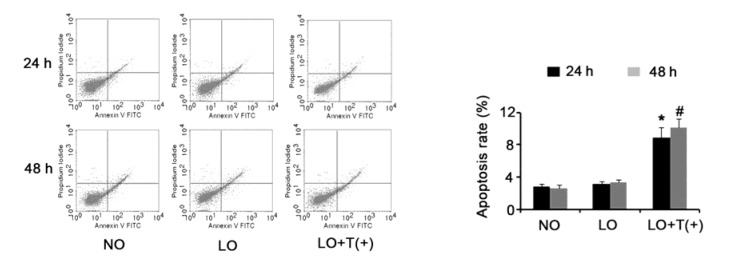
AEG-1 knockdown increased apoptosis rate of HepG2 in hypoxia HepG2 cells with AEG-1 knockdown or not were incubated in hypoxia for 24 or 48 h, before apoptosis rate dectection via flow cytometry. Apoptosis rate of HepG2 cells was not altered after culture in hypoxia for 24 or 48 h, compared to apoptosis rate of the cells cultured in normoxia. However, HepG2 cells with AEG-1 knockdown showed dramatically elevated apoptosis rate following incubation in hypoxia for 24 and 48 h. NO: normoxia; LO: low oxygen; LO + T(+) : HepG2 cells transfected with shRNA-746 were incubated in hypoxia. *P < 0.05 and #*P* < 0.05 vs. NO, n = 4.

**Figure 6:**
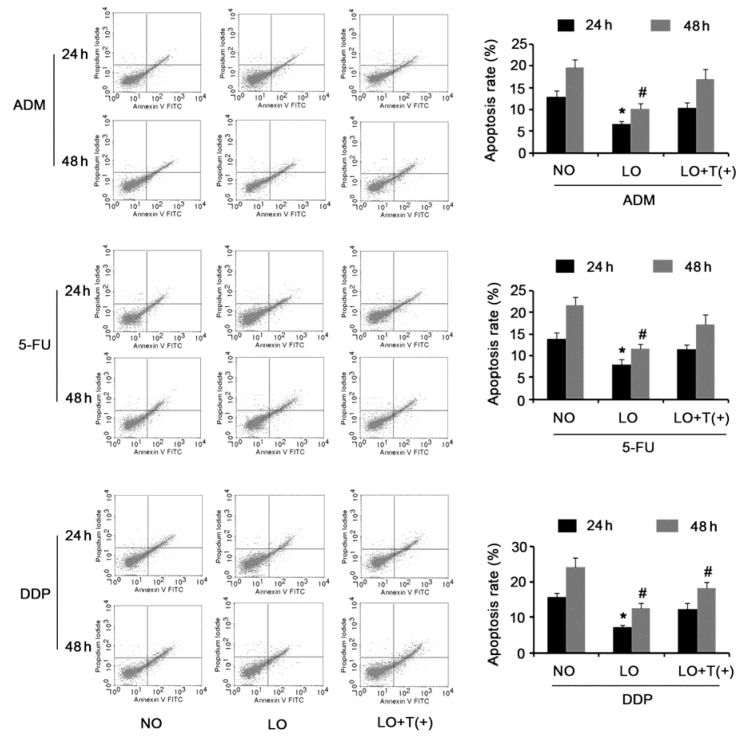
AEG-1 knockdown enhanced apoptosis-induced ability of ADM, 5-FU and DDP to HepG2 cells in hypoxia HepG2 cells with AEG-1 knockdown or not were incubated with 200 ng/ml ADM, 50 μg/ml 5-FU, or 25 μg/ml DDP in hypoxia for 24 or 48 h, before apoptosis rate dectection via flow cytometry. NO: normoxia; LO: low oxygen; LO + T(+) : HepG2 cells transfected with shRNA-746 were incubated in hypoxia. *P < 0.05 and #*P* < 0.05 vs. NO, n = 4.

Apoptosis assay demonstrated that apoptosis rate of HepG2 cells was not altered after culture in hypoxia for 24 or 48 h, compared to apoptosis rate of HepG2 cells cultured in normoxia (Figure 5(Fig. 5)). However, HepG2 cells with AEG-1 knockdown showed dramatically elevated apoptosis rate following incubation in hypoxia for 24 (*P* = 0.027) or 48 h (*P* = 0.019). Incubation in hypoxia for 24 (*P* = 0.034) or 48 h (*P* = 0.029) significantly inhibited the apoptosis that induced by 200 ng/ml ADM, compare to incubation in normoxia (Figure 6(Fig. 6)). AEG-1 knockdown in HepG2 cells abolished the apoptosis-inhibitory effect induced by hypoxia. Incubation in hypoxia for 24 (*P* = 0.039) or 48 h (*P* = 0.033) also resulted in remark reduction in the apoptosis rate triggered by 50 μg/ml 5-FU, while this inhibitory effect on apoptosis was abrogated with AEG-1 depletion. Apoptotic response induced by 25 μg/ml DDP was blunted with HepG2 incubated in hypoxia for 24 (*P* = 0.017) or 48 h (*P* = 0.024). Silencing AEG-1 to some extent recovered the sensitivity of HepG2 to DDP in hypoxia for 24 (*P* > 0.05) or 48 h (*P* = 0.041). 

### PI3K/AKT/HIF-1α/MDR-1 signaling pathway was regulated by AEG-1

**Figure 7:**
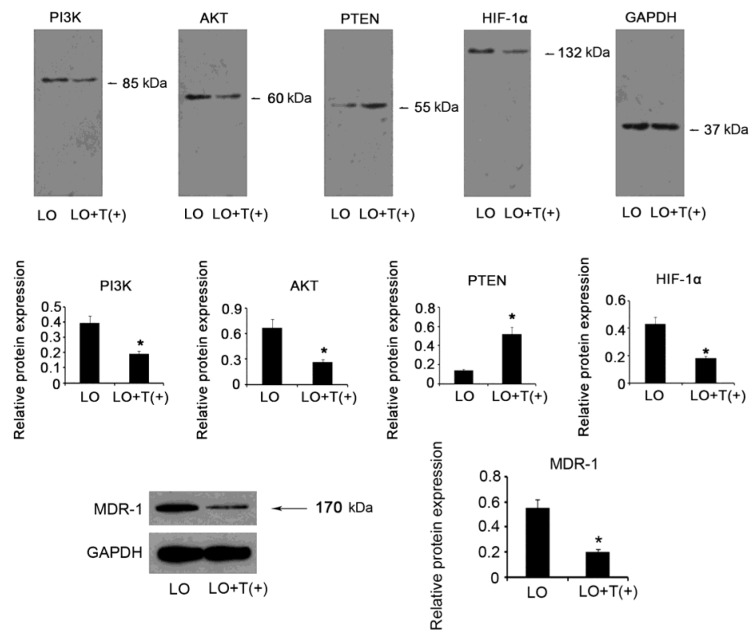
PI3K/AKT/HIF-1α/MDR-1 signaling pathway was regulated by AEG-1 HepG2 cells with AEG-1 knockdown or not were incubated in hypoxia for 48h, followed by examination of protein expression levels of PI3K, AKT, HIF-1α, PTEN, and MDR-1 via Western blot assay. HIF-1α:hypoxia-inducible factor 1; PTEN: phosphatase and tensin homolog; PI3K: phosphatidylinositol 3-kinase; MDR-1: multiple drug resistance gene-1. Proteins were separated on a 12% SDS-PAGE gel, excepted for MDR-1 detection that the proteins were separated on a 10% SDS-PAGE gel. LO: low oxygen; LO + T(+) : HepG2 cells transfected with shRNA-746 were incubated in hypoxia. *P < 0.05 vs. LO, n = 4.

Previous researches document that signaling pathway mediated by PI3K/AKT/HIF-1α/MDR-1 plays critical role in hypoxia-induced chemoresistance. To determine whether these signaling molecules are regulated by AEG-1 in hypoxia, we knocked AEG-1 down and evaluated their protein expression after incubation of the cells in hypoxia for 48 h. AEG-1 depletion resulted in dramatic reduction in protein expression of PI3K (*P* = 0.042), AKT(*P* = 0.038), HIF-1α (*P* = 0.043) and MDR-1 (*P* = 0.034), while PTEN expression (*P* = 0.028) was increased with AEG-1 knockdown (Figure 7(Fig. 7)). It indicates PI3K/AKT/HIF-1α/MDR-1 pathway is positively regulated by AEG-1. 

## Discussion

AEG-1 has been verified to be up-regulated in HCC and associated with the development and progression (Yoo et al., 2009[30]; Robertson et al., 2015[21]). AEG-1 is up-regulated in various HCC cell lines compared to the normal counterparts (Ma et al., 2014[19]). HCC tissue samples also have higher AEG-1 expression than normal liver samples, as demonstrated by the present study. Importantly, AEG-1 expression in HCC is increased with the stages from I to IV as well as grades of the differentiation from well differentiated to poorly differentiated, which suggests AEG-1 is positively correlated with HCC development and metastasis (Ahn et al., 2013[1]; Li et al., 2015[16]). Study performed in transgenic mice showed that the hepatocyte-specific expression of AEG-1 give rise to the generation of multinodular HCC with steatotic features following treatment with N-nitrosodiethylamine, but this event was not observed in wild type mice (Srivastava et al., 2012[22]). Hepatocytes isolated from the AEG-1 transgenic mice exhibited profound resistance to cell death induced by DNA damage, growth factor deprivation and chemotherapeutics (Srivastava et al., 2012[22]). Microarray analysis indicates that AEG-1 is involved in the regulation of genes associated with proliferation, invasion, metastasis, chemoresistance, angiogenesis and senescence (Meng et al., 2013[20]). 

Although AEG-1 serves as an important cancer promoter in HCC, but its effect on modulation of chemoresistance in hypoxia is not understood. Hypoxia is a common event in HCC and other solid tumors, due to rapid growth of tumor cells that outstrips its vasculature as well as structural and functional abnormality of newly formed intra-tumoral vessels (Flamant et al., 2012[7]; Bogaerts et al., 2015[2]). Evidence is mounting that HCC in hypoxic regions harbors strong resistance to multiple cytotoxic agents, which presents a clinical challenge in chemotherapy (Zhang et al., 2016[32]; Flamant et al., 2012[7]; Bogaerts et al., 2015[2]). Study, herein, also demonstrated that hypoxia blunted responses of HCC HepG2 cells to ADM, 5-FU and DDP, as evidenced by cell viability and apoptosis assays. In addition, the present study for the first time found that AEG-1 expression in HepG2 cells was increased in hypoxia when compared to that in normoxic circumstance; deletion of AEG-1 via shRNA interference enhanced the sensitivity of HepG2 cells to these agents in hypoxia, which indicates that AEG-1 functions as an important mediator responsible for chemoresistance of HepG2 cells in hypoxia. 

Numerous studies have established the functional role of MDR-1 in tumor chemoresistance (Hyuga et al., 2012[9]). MDR-1 belongs to the ATP-binding cassette (ABC)-transporter family and contributes to multidrug resistance through the efflux of a wide range of chemotherapeutic drugs from cancer cells (Hyuga et al., 2012[9]). MDR-1 over-expression is commonly associated with a low chemotherapeutic efficiency and poor prognosis of cancers, while downregulation of MDR-1 expression is an effective way to enhance the chemosensitivity of cancer cells to conventional chemotherapeutic agents. Mounting evidence indicates that hypoxia is an important incentive factor for MDR-1 expression. Yang et al. (2016[28]) showed MDR-1 expression is up-regulated in colorectal cancer LOVO cells in CoCl₂-induced hypoxia (Yang et al., 2016[28]). In addition, MDR-1 expression in laryngeal cancer cells is increased following exposure to hypoxic environment (Li et al., 2016[15]). Herein, we observed that incubation of HCC cells in hypoxia also gave rise to up-regulation of MDR-1 in both mRNA and protein levels, thus we suggested that MDR-1 regulation mediated the actions of hypoxia-induced chemoresistance. 

Bioactivities of HIF-1α are related to the oxygen concentration in surrounding environment and relevant signaling pathway. Hypoxia has been identified as a predominant cause to HIF-1α activation (Li et al., 2015[18]). Since HIF-1α is a key up-stream modulator of MDR-1, it is easy to understand that MDR-1 expression was raised under hypoxia (Zhan et al., 2015[31]). HIF-1α abundance and activity are also regulated by PI3K/AKT signaling pathway. Considering there is a crosstalk between AEG-1 and PI3K/AKT signal, we hypothetise that AEG-1 may be implicated in the regulation of HIF-1α and its downstream target MDR-1 (Jiao and Nan, 2012[10]). The present study showed that PI3K and AKT expression were down-regulated with AEG-1 knockdown, but their inhibitor, PTEN, was up-regulated. It indicates that AEG-1 exerts promoting effects on PI3K/AKT signal in hypoxic HCC cells. As we expected, attenuated PI3K/AKT signal that was resulted from AEG-1 depletion resulted in inhibition of HIF-1α/MDR-1 pathway, thus it is suggested that AEG-1 positively modulated MDR-1 expression in hypoxic HCC via the stimulation of PI3K/ AKT/HIF-1α signaling pathway.

In conclusion, this study for the first time presented evidence that AEG-1 is associated with hypoxia-induced HCC chemoresistance. Further, we revealed that the AEG-1 stimulating PI3K/AKT/HIF-1α/MDR-1 is underlying the mechanisms by which AEG-1 promotes the chemoresistance. This study contributes to the target therapy that overcomes the multidrug resistance in HCC.

## Acknowledgements

The authors thank Professor De-Wu Zhong for his valuable suggestions and critical revision of the manuscript.
